# Finding the pond through the weeds: eDNA reveals underestimated diversity of pondweeds

**DOI:** 10.1002/aps3.1155

**Published:** 2018-06-05

**Authors:** Maria L. Kuzmina, Thomas W. A. Braukmann, Evgeny V. Zakharov

**Affiliations:** ^1^ Centre for Biodiversity Genomics University of Guelph 50 Stone Road East Guelph Ontario N1G2W1 Canada

**Keywords:** aquatic plants, *atpB‐rbcL*, eDNA metabarcoding, ITS2, Ontario, Potamogetonaceae

## Abstract

**Premise of the Study:**

The detection of environmental DNA (eDNA) using high‐throughput sequencing has rapidly emerged as a method to detect organisms from environmental samples. However, eDNA studies of aquatic biomes have focused on surveillance of animal species with less emphasis on plants. Pondweeds are important bioindicators of freshwater ecosystems, although their diversity is underestimated due to difficulties in morphological identification and monitoring.

**Methods:**

A protocol was developed to detect pondweeds in water samples using *atpB*‐*rbcL* and ITS2 markers. The water samples were collected from the Grand River within the rare Charitable Research Reserve, Ontario (RARE). Short fragments were amplified using primers targeting pondweeds, sequenced on an Ion Torrent Personal Genome Machine, and assigned to the taxonomy using a local DNA reference library and GenBank.

**Results:**

We detected two species earlier documented at the experimental site during ecological surveys (*Potamogeton crispus* and *Stuckenia pectinata*) and three species new to the RARE checklist (*P. foliosus*,* S. filiformis*, and *Zannichellia palustris*).

**Discussion:**

Our targeted approach to track the species composition of pondweeds in freshwater ecosystems revealed underestimation of their diversity. This result suggests that eDNA is an effective tool for monitoring plant diversity in aquatic habitats.

Environmental DNA (eDNA) is ubiquitous in water, aquatic sediments, and soil. Live and dead single‐celled organisms, extracellular secretions, gametes, blood, spores, and pollen are all sources of DNA that can be detected in aquatic environments. The detection of eDNA with high‐throughput sequencing technologies is a rapidly emerging approach to document the occurrence of organisms (Shaw et al., 2016; Deiner et al., 2017). To date, it has primarily focused on the surveillance of animal species, such as freshwater fish and amphibians that are invasive, rare, or difficult to monitor (Dejean et al., 2012; Sigsgaard et al., 2015; Biggs et al., 2015). The detection of plant eDNA in aquatic and soil habitats is understudied (Scriver et al., 2015; Fahner et al., 2016).

The pondweed family (Potamogetonaceae) is a cosmopolitan group of aquatic angiosperms from the order Alismatales (APG IV, 2016) with high species diversity in the Northern Hemisphere (Lindqvist et al., 2006). In North America, this family is represented by three genera and 37–42 species: *Potamogeton* L. (32–37), *Stuckenia* Börner (4), and *Zannichellia* L. (1) (Haynes and Hellquist, 2000; Ulloa et al., 2017). Pondweeds are vital macrophytes in freshwater ecosystems, providing food and shelter for fish, birds, macroinvertebrates, and plankton (Engel, 1988; Dibble and Harrell, 1997; Sandilands, 2005). Species‐specific affiliation with freshwater habitats makes them important bioindicators that are useful for the classification of the aquatic plant communities and the evaluation of water quality (Holmes et al., 1998; Peng et al., 2008; Lukacs et al., 2009). Pondweeds vary in their adaptations to chemical composition, temperature, and the flow rate of water (Lusa et al., 2011; Robionek et al., 2015). For example, *S. pectinata* (L.) Börner often colonizes disturbed and heavily polluted aquatic communities (Dixon et al., 2006), whereas in contrast, other species such as *P. friesii* Rupr. and *P. hillii* Morong are inhabitants of clear calcareous waters (Gleason and Cronquist, 1991). Seven species of pondweeds are considered endangered or recognized as species at risk in North America (U.S. Fish and Wildlife Service, 2018). Two of these, *P. hillii* (COSEWIC, 2005) and *P. ×ogdenii* Hellq. & R. L. Hilton (COSEWIC, 2007), are found in Ontario. The federal recovery strategy for these two species called for surveys to reconfirm their presence at previously reported locations and identify new occurrence sites within their distribution (Parks Canada, 2012; Environment Canada, 2015).

The morphological identification of pondweeds is often limited by phenology (during fruiting period) and microscopic traits (Fernald, 1932). Therefore, species‐level identification of pondweeds is difficult for non‐experts who often assist with field work. Additionally, aquatic habitats are often less accessible than terrestrial ones, with many plant species being completely submersed, and thus difficult to find. Overall, these factors lead to sporadic, incomplete records for aquatic macrophytes in ecological surveys and inventories (Wetzel, 1983). A targeted metagenomic approach for detection of this group of aquatic plants has the potential to overcome difficulties with their monitoring and identification during ecological surveys.

We explored the detection of pondweeds using eDNA in water samples collected along the Grand River, Ontario, Canada, within the rare Charitable Research Reserve (RARE). We further compared our results with the checklist of RARE that was generated using traditional methods of collecting and morphological identification. Finally, we tested how the markers from different genome compartments (plastid and nuclear), marker length, and primer specificity affect the taxonomic assignment of the eDNA fragments to the species of pondweeds.

## MATERIALS AND METHODS

### Selecting eDNA markers and creating a DNA reference library

Using tissue and DNA available within the Centre for Biodiversity Genomics (CBG) archive, we selected 30 species of pondweeds recorded in Ontario: *Potamogeton* (26), *Stuckenia* (3), and *Zannichellia* (1) (Brouillet et al., 2010; Desmet and Brouillet, 2013; dx.doi.org/10.5883/DS‐VASCAN). Species of known hybrid origin (e.g., *P. ×ogdenii*), defined in the literature as nothospecies (McNeill et al., 2012), were not included in the list. Each species was represented on average by three specimens with verified taxonomy (90 specimens) (dx.doi.org/10.5883/DS‐POTAM) and used to generate the local pondweed reference library (DS‐POTAM). DNA was extracted with the standard CBG protocols (Ivanova et al., 2008, 2011). Two highly variable DNA regions were selected to design eDNA markers: the plastid‐encoded intergenic spacer *atpB*‐*rbcL* (Ito et al., 2014) and the nuclear‐encoded ribosomal internal transcribed spacer ITS2 (China Plant BOL Group, 2011). The primers for these markers, specific for Potamogetonaceae, were designed to recover all species selected for the local reference library that generated amplicons suitable for eDNA detection.

The primary data sets included sequences belonging to three North American genera (*Potamogeton*,* Stuckenia*, and *Zannichellia*) downloaded from GenBank (*atpB*‐*rbcL*: AB871483–AB871490) and from the Barcode of Life Data management systems (ITS2: BOLD: dx.doi.org/10.5883/DS‐VASCAN; Ratnasingham and Hebert, 2007). The data sets included a ~740–770‐bp region for *atpB*‐*rbcL* (Appendix S1, A: primers 1–2; Manen et al., 1994) and a ~360–490‐bp region for ITS2 (Appendix S1, A: primers 3–5; White et al., 1990; Chen et al., 2010). Sequences were aligned with MAFFT (Katoh et al., 2002) and used to manually design primers with a high specificity for Potamogetonaceae. Although these primers should preferentially bind Potamogetonaceae species, many priming regions are conserved, and will potentially amplify non‐target species (i.e., Cheng et al., 2016). The annealing temperature, self‐dimerization, hairpin formation, and self‐annealing parameters for the primers were checked with Oligo Calculator version 3.27 (Kibbe, 2007).

The primers successfully amplifying pondweeds (Appendix S1, A: primers 6–9) were then used to build a complete reference DNA barcode library for 30 species of pondweeds recorded in Ontario. Amplicons were obtained using the protocols described in Appendix S1 (B and C), sequenced following standard procedures for the ABI 3730*xl* DNA Analyzer (Applied Biosystems, Foster City, California, USA), and uploaded to BOLD. Newly generated data sets included 71 *atpB*‐*rbcL* sequences (mean length 450 bp) for 28 species and 88 ITS2 sequences (mean length 260 bp) (Appendix S2). The generated DNA barcode reference libraries for *atpB*‐*rbcL* and ITS2 were used to design shorter markers for amplifying degraded DNA present in environmental samples (Table 1; Appendix S1, A: primers 10–14). Based on the reference libraries, two markers of different length for *atpB*‐*rbcL* (117 bp and 184 bp) were used to explore the influence of amplicon length on species detection, along with a 157‐bp region of ITS2 as eDNA markers.

**Table 1:** eDNA markers used to detect species of pondweeds in water samples. The range of possible lengths (bp) and mean lengths (bp) are indicated for the species included in the local reference library

eDNA marker	Length (bp)	Mean length (bp)	Primer pairs^a^
atpB‐rbcL‐117	101–133	117	10 and 12
atpB‐rbcL‐184	161–208	184	11 and 12
ITS2‐157	133–182	157	13 and 14

a Primer pairs referenced in Appendix S1, A; these were used to amplify eDNA markers in the first round of PCR (PCR1).

**Figure 1:**
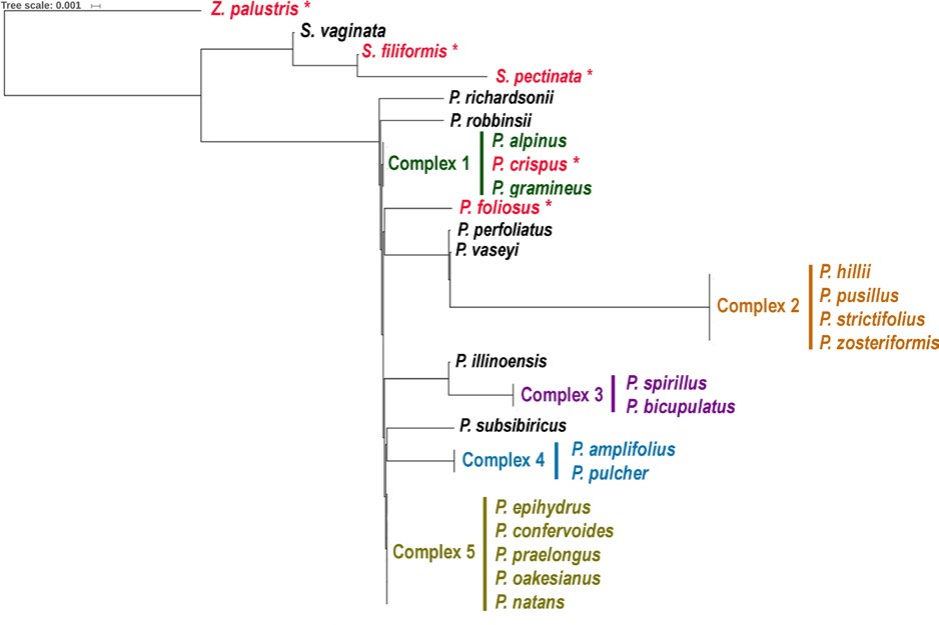
Phylogenetic tree modeling species resolution for eDNA marker atpB‐rbcL‐117 (mean length 117 bp). Unresolved groups of species are labeled as complexes. Trees were generated in Fast Tree (Price et al., 2010).

**Figure 2:**
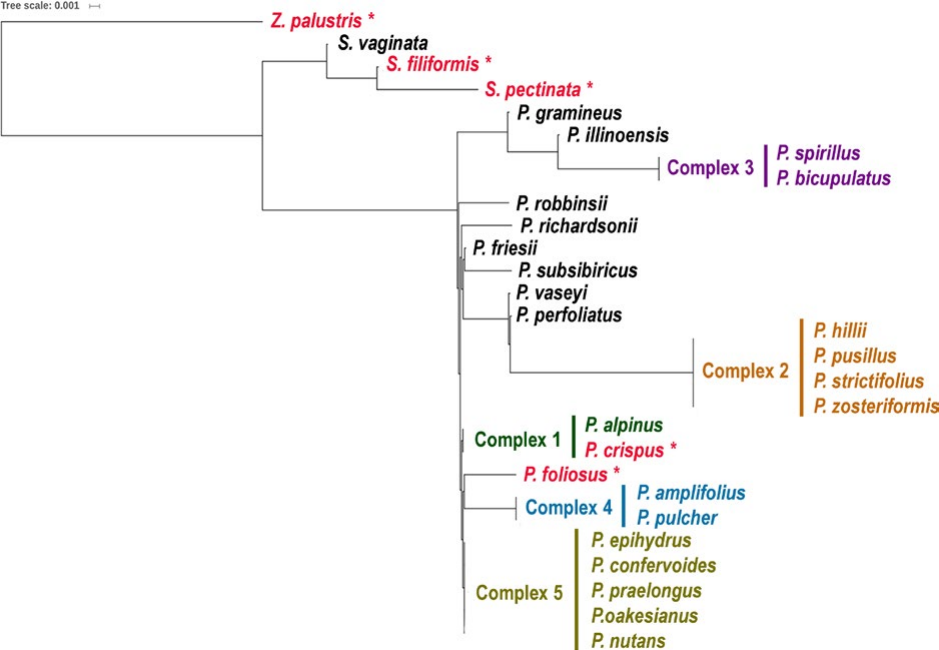
Phylogenetic tree modeling species resolution for eDNA marker atpB‐rbcL‐184 (mean length 184 bp). Unresolved groups of species are labeled as complexes. Trees were generated in Fast Tree (Price et al., 2010).

**Figure 3:**
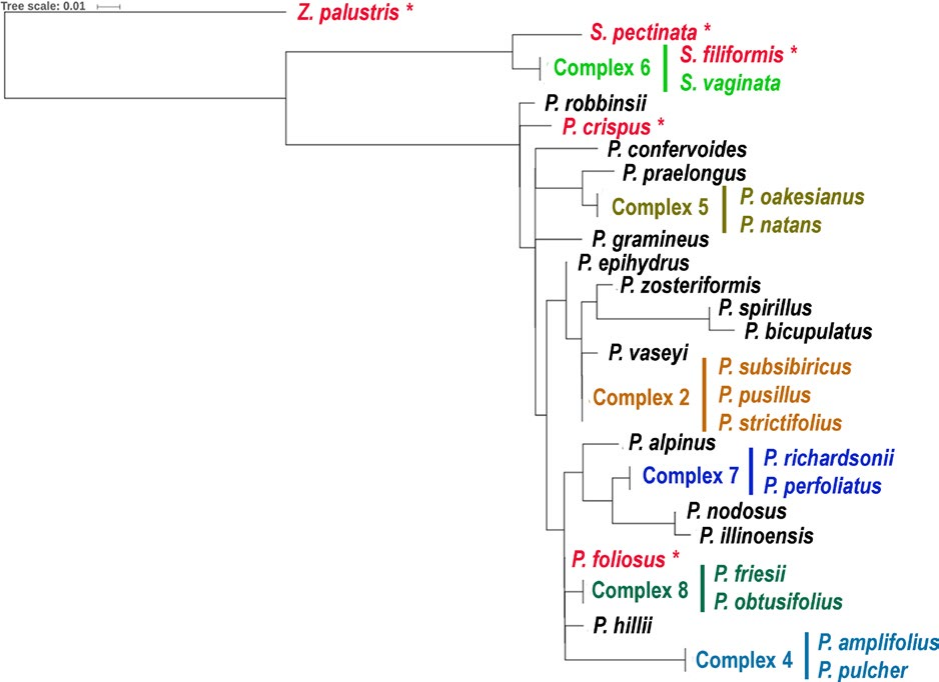
Phylogenetic tree modeling species resolution for eDNA marker ITS2‐157 (mean length 157 bp). Unresolved groups of species are labeled as complexes. Trees were generated in Fast Tree (Price et al., 2010).

To model species resolution for Ontario pondweeds for the eDNA markers, one individual per species was selected, as no intraspecific variation was observed within the generated alignments. Each of the three fragments were analyzed using distances calculated with the Tamura–Nei model in Geneious version 9.1.5 (Tamura and Nei, 1993) and approximated with a maximum likelihood tree using the Fast Tree algorithm (Price et al., 2010) (Figs. 1, 2, 3, Appendix S3). The groups of unresolved species were indicated in the local reference libraries as “complexes.”

### Selection of the field site

The selected site is along the Grand River within RARE (43.3859, −80.3717) where two species of pondweeds (*S. pectinata* and *P. crispus* L.) were previously documented (Telfer et al., 2015). Herbarium vouchers were identified and deposited at the BIO Herbarium, University of Guelph (OAC; acronym used in accordance with Index Herbariorum [Thiers, 2017]). Tissue and DNA from the specimens are stored at the Center for Biodiversity Genomics (BIOUG24048‐C12, BIOUG24048‐D12); DNA barcodes are available on BOLD (dx.doi.org/10.5883/DS‐POTAM).

### Collecting water samples and eDNA extraction

**Figure 4:**
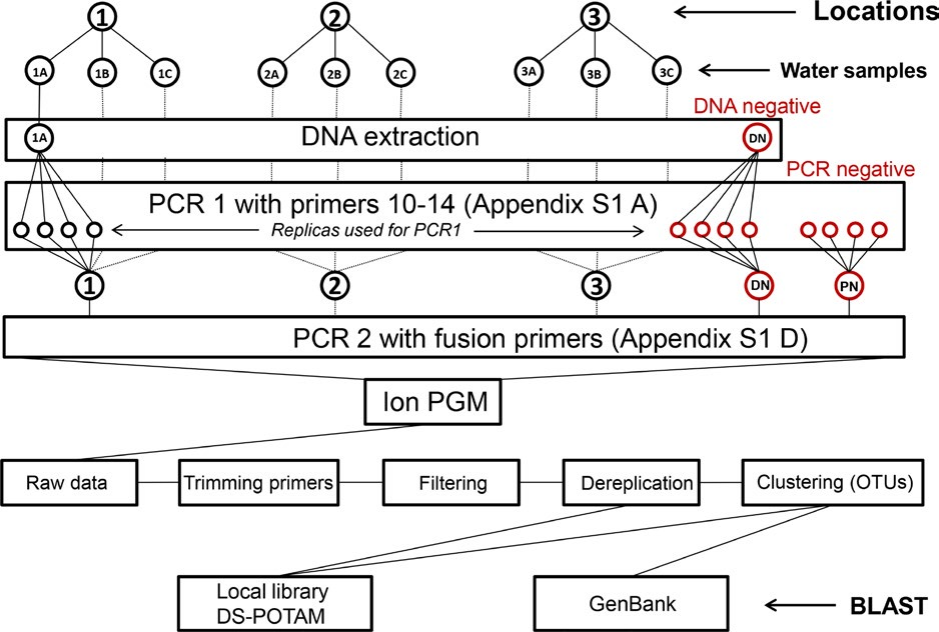
The workflow of the experiment starting with the collection of water samples from three locations along the Grand River, Ontario, Canada. Collection was followed by laboratory work: eDNA extraction (including DNA negative control), amplification of the target eDNA markers from the samples and controls with regular primers, pooling of PCR1 products, labeling pooled products via PCR2 with Ion Personal Genome Machine MID tags using fusion primers, and sequencing on the Ion Torrent Personal Genome Machine. The bioinformatic workflow included trimming primers, filtering, dereplication, and clustering followed by BLAST searches against the local reference library (BOLD: DS‐POTAM) and GenBank.

Water samples were collected from three locations 200 m apart from each other, on 8 September 2016. Three replicates of 0.5 L of water were sampled in sterile plastic bottles from each location (Fig. 4). The samples of water were transported in a cooler to the laboratory and stored at 4°C overnight. Samples were filtered through MicroFunnel 0.2‐μm water filters (Pall Corporation, Port Washington, New York, USA). After filtration, each membrane was transferred to PowerWater DNA Isolation Kit tubes (catalog no. 14900‐50‐NF; MO BIO Laboratories, Carlsbad, California, USA) and stored at 4°C overnight. DNA from the membranes was then extracted using the PowerWater DNA Isolation Kit.

### High‐throughput sequencing strategy

Each of the nine DNA samples, one negative DNA control, and one negative PCR control were amplified in four replicates (Fig. 4). The first round of PCR was performed with primers 10–14 (Appendix S1). Prior to the second round of PCR, replicates were pooled and labeled with IonExpress MID tags (Thermo Fisher Scientific, Waltham, Massachusetts, USA) using fusion primers to produce barcoded amplicon libraries (Appendix S1, B–D). The fusion primers used in this study targeted the priming region with a tail containing P1 ISP binding adapter, key, and IonExpress MID tags for the reverse primers and the trP1 adapter for the forward primers (Appendix S1, D). All labeled products were pooled and the sequencing library was prepared with the Ion PGM Hi‐Q OT2 400 Kit and the Ion PGM Hi‐Q Sequencing Kit (Thermo Fisher Scientific) according to the manufacturer’s instructions. All products were sequenced using an Ion 318 v2 chip on the Ion Torrent Personal Genome Machine (PGM; Thermo Fisher Scientific).

### Bioinformatic workflow

The raw sequencing data were subject to two different bioinformatics pipelines (Fig. 4). The first one compared dereplicated sequences (FASTX Toolkit: http://hannonlab.cshl.edu/fastx_toolkit/) with the local reference libraries for pondweeds for *atpB*‐*rbcL* (28 species) and ITS2 (30 species) using BLAST through QIIME (Caporaso et al., 2010). This analysis was performed to differentiate and detect species with minor distances (1–2 bp). The second approach included generation of operational taxonomic units (OTUs) with 99% identity using Uclust version 1.2.22q (Edgar, 2010). The OTUs were similarly analyzed using a pondweed reference library, and the results were compared with those obtained through the analysis of dereplicated sequences. To indicate taxonomic affiliation with the non‐target organisms (non‐pondweeds), the OTUs were compared with a global reference library (GenBank). Both pipelines included trimming of the primer sequences using Cutadapt version 1.8.1 (Marcel, 2011) and filtering based on quality (QV20) and length (minimum 100 bp) with Sickle version 1.33 (Joshi and Fass, 2011). Additional filtering criteria were applied to exclude sequences with (1) less than 98% identity of a query sequence to a reference, (2) lower than two thirds overlap calculated based on the average expected length of the amplified DNA fragments (i.e., 77 bp for 117‐bp fragment of *atpB*‐*rbcL*, 121 bp for 184‐bp fragment of *atpB*‐*rbcL*, 104 bp for 157‐bp fragment of ITS2), and (3) threshold more than 10 reads assigned to a taxon in the reference library.

## RESULTS

### Species resolution for the pondweeds in Ontario with eDNA markers

Two eDNA markers from the *atpB*‐*rbcL* region (atpB‐rbcL‐117 and atpB‐rbcL‐184) had similar species resolution, discriminating 12–13 species out of 28 reference species (Figs. 1, 2; Appendix S3). The unresolved clades (complexes) contained 3–5 species (complexes 1, 2, and 5) or two species (complexes 3 and 4). The ITS2‐157 region resolved 17 of 30 species (Fig. 3, Appendix S3). Only complex 2 included three species, whereas complexes 4–8 were formed by pairs of closely related species. The combination of all three eDNA markers succeeded in resolving 23 species of pondweeds out of 30 references.

### Taxonomic assignment of dereplicated sequences and OTUs

**Figure 5:**
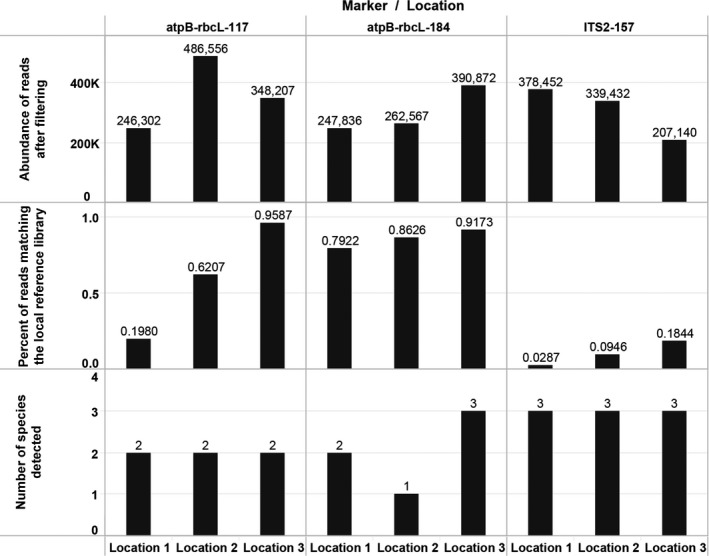
The abundance of reads after filtering, proportion of reads matching pondweeds, and the number of the detected species for three eDNA markers (atpB‐rbcL‐117, atpB‐rbcL‐184, and ITS2‐157) from three locations.

Abundance of reads after filtering for three markers (atpB‐rbcL‐117, atpB‐rbcL‐184, and ITS2‐157) varied between 200,000–500,000 for all locations (Fig. 5). The number of dereplicated sequences and OTUs associated with atpB‐rbcL‐117 and atpB‐rbcL‐184 was on average substantially lower (8000 dereplicated sequences, 110 OTUs) compared to ITS2‐157 (70,000 dereplicated sequences, 8500 OTUs) (Appendix S4). By contrast, the proportion of reads assigned to the target DNA (species of pondweeds represented in the local DNA reference library) was on average the highest for atpB‐rbcL‐184 (85%), followed by atpB‐rbcL‐117 (59%), and the lowest for ITS2‐157 (10%) (Fig. 5). The low number of hits to target species for ITS2‐157 indicates substantial co‐amplification of non‐target DNA. Despite the lower specificity of ITS2‐157, three species of pondweeds were detected at each location, whereas atpB‐rbcL‐117 and atpB‐rbcL‐184 on average detected two species at each of the locations (Fig. 5).

Five detected taxa were resolved to species level using our custom libraries, supported with more than one marker and/or in multiple locations (*P. crispus*,* P. foliosus* Raf., *S. filiformis* (Pers.) Börner, *S. pectinata*, and *Z. palustris* L.) (Table 2). BLAST results for both data sets (dereplicated sequences and OTUs) using a pondweed reference library showed overall consistency among all eDNA markers and across all locations. An exception was the discrimination of *S. filiformis* from *S. pectinata* by the dereplication data sets for atpB‐rbcL‐117 at all three locations. The minor distance between these two species (two base pairs: Appendix S3, A) was masked by the clustering approach for tracking pondweed diversity, which grouped reads belonging to closely related species. Twelve dereplicated sequences with atpB‐rbcL‐184 were assigned to *S. vaginata* (Turcz.) Holub only in the third location.

**Table 2:** Number of reads associated with the detected taxa in the eDNA samples (three locations, DNA negative, and PCR negative). Results are provided separately for each of the bioinformatic pipelines (dereplicated sequences and operational taxonomic units [OTUs]).^a^

Top hit identification^b^	Locations and negative controls	Library	eDNA marker/Raw data treatment					
			atpB‐rbcL‐117		atpB‐rbcL‐184		ITS2‐157	
			Dereplicated	OTUs	Dereplicated	OTUs	Dereplicated	OTUs
Target DNA (Potamogetonaceae)								
*Potamogeton crispus*	Location 2	DS‐POTAM					270	275
		GenBank						275
*Potamogeton foliosus*	Location 1	DS‐POTAM					1363	1367
	Location 2	DS‐POTAM					454	453
	Location 3	DS‐POTAM			14,062	13,954	1267	1274
***Potamogeton gayi***	Location 3	GenBank				**13,938**		
***Potamogeton pusillus***	Location 1	GenBank						**1367**
	Location 2	GenBank						**453**
	Location 3	GenBank						**1274**
*Stuckenia filiformis*	Location 1	DS‐POTAM	778					
	Location 2	DS‐POTAM	6412					
	Location 3	DS‐POTAM	4031					
*Stuckenia pectinata*	Location 1	DS‐POTAM	47,979	49,141	191,594	180,965	9263	9267
		GenBank		49,141		180,967		9267
	Location 2	DS‐POTAM	295,614	307,529	226,484	212,250	30,684	30,775
		GenBank		307,779		212,272		30,775
	Location 3	DS‐POTAM	329,810	335,372	344,475	324,822	34,610	34,639
		GenBank		335,372		324,824		34,639
	PCR negative	DS‐POTAM			12	12		
		GenBank				12		
*Stuckenia vaginata*	Location 3	DS‐POTAM			12			
*Zannichellia palustris*	Location 1	DS‐POTAM			4750	5482	227	228
		GenBank				5482		228
	Location 2	DS‐POTAM					697	698
		GenBank						698
	Location 3	DS‐POTAM					2322	2320
		GenBank						2320
Non‐target DNA								
*Panicum capillare*	Location 2	GenBank						34
Charophyta	Location 1	GenBank						25
Cyanobacteria	Location 1	GenBank						60
	Location 2	GenBank						13
Proteobacteria	Location 1	GenBank						229
	Location 2	GenBank		28				153
	Location 3	GenBank						40
	DNA negative	GenBank						77

a The number of reads associated with two species identified using the GenBank library (*P. gayi* and *P. pusillus*, in bold) corresponds with the number of reads associated with *P. foliosus* identified using the local pondweed reference library.

b The top hits were identified with the local pondweed reference library (DS‐POTAM) for both dereplicated sequences and OTUs. The latter were also compared with the public nucleic acid repository (GenBank).

Only OTUs were compared with the global reference library (GenBank) to summarize the higher‐level taxonomy of non‐target (i.e., non‐pondweed) sequences. The BLAST results recovered *S. pectinata* and *Z. palustris* in all locations for both *atpB‐rbcL* and ITS2 eDNA markers with similar read depth (Table 2). The presence of *P. crispus* was also confirmed by GenBank data for ITS. In contrast, the sequences assigned with the local reference library to *P. foliosus* (100% identity to MF694340) in GenBank were identical to the specimen identified as *P. pusillus* L. collected from Turkey (KX273110). OTUs for atpB‐rbcL‐117 and atpB‐rbcL‐184 assigned to *P. foliosus* (100% identity to MF694509) with the local library matched *P. gayi* A. Benn. (a tropical species from South America commonly cultivated in aquariums) in GenBank (98% identity to KT634258).

In addition to the species of pondweeds, the GenBank reference data for ITS indicated only one species of flowering plants in the second location (*Panicum capillare* L. [34 reads, 1 OTU], a common weed in North America) (Table 2). The same database indicated presence of Charophyta (location 1), Cyanobacteria (locations 1 and 2), and Proteobacteria (all three locations).

The DNA negative control contained 77 identical sequences (one OTU) of ITS2‐157 assigned to Proteobacteria (*Comamonas aquatica*) with 98% identity (CP016603). The PCR negative control for atpB‐rbcL‐184 had 12 reads of *S. pectinata*.

## DISCUSSION

ITS2‐157 showed overall higher species resolution (57%) compared to both *atpB‐rbcL* eDNA markers (43–46%) despite a lower proportion of reads matching the local reference library. The higher resolution of ITS2 relative to the *atp‐rbcL* markers is a result of the better taxonomic resolution of pondweed species by ITS2. The complexes of unresolved species with ITS2‐157 included 2–3 closely related species, whereas *atpB‐rbcL* showed polytomy for the groups of 2–5 species (Figs. 1, 2, 3). In addition to the “linear‐leaved lineage” (complex 2), which is known for being challenging to discriminate morphologically (Haynes and Hellquist, 2000; Lindqvist et al., 2006), *atpB‐rbcL* failed to identify the “broad‐leaved” species of pondweeds, although they have more distinct morphological characteristics (complexes 1, 4, and 5). However, the species‐level resolution was complemented by two genome compartments (chloroplast and nuclear) resulting in discrimination of 77% of the pondweed species from Ontario. For example, the lack of resolution between *S. vaginata* and *S. filiformis* with ITS2 (complex 6) was redeemed with *atpB‐rbcL*, which discriminated these two species. Conversely, ITS2 unambiguously resolved *P. crispus* and *P. hillii* (complexes 1 and 2 with *atpB*‐*rbcL*). Five species of pondweeds detected in our experiment were resolved by at least one of the three eDNA markers (Figs. 1, 2, 3, species in red font with asterisk).

The three markers tested in this experiment (atpB‐rbcL‐117, atpB‐rbcL‐184, and ITS2‐157) demonstrated suitability for detection of eDNA extracted from water samples. The performance of the plastid markers was highly specific, targeting the species from Potamogetonaceae almost exclusively, especially the longer *atpB‐rbcL* fragment (85% average). Despite ITS2 showing a lower ratio of reads matching the reference library (10%), it detected a relatively broader range of species from this group, compared to *atpB*‐*rbcL* markers (Table 2, Fig. 5). The high copy number per compartment, and the less permeable membrane of chloroplasts and mitochondria relative to the nucleus, suggest that organellar DNA persists longer in the environment than nuclear DNA (Nielsen et al., 2007; Barnes and Turner, 2016). The lower number of well‐preserved copies of nuclear DNA relative to cpDNA, in combination with more conserved priming regions for ITS2, may explain the observed co‐amplification of non‐target taxa. However, only a small proportion of the non‐target ITS2 reads (~0.06%) were identified using a BLAST algorithm as different algal and microbial communities in the analyzed locations. Between two chloroplast markers, the shortest one (mean 117 bp) succeeded in consistent detection of *S. filiformis* in all three locations, whereas the longest marker (mean 184 bp) failed to do so. We suggest this species likely occurred farther from the experimental site, and was therefore represented by more degraded eDNA, compared to *S. pectinata*. The use of longer markers may indicate the location of target populations more accurately because the extent of DNA degradation increases with distance from source specimens (Seymour et al., 2018).

In addition to two species previously documented at RARE (*S. pectinata* and *P. crispus*), we detected three species (*P. foliosus*,* S. filiformis*, and *Z. palustris*) that are new to the experimental site and not included in the checklist for this nature reserve. Our findings are supported by the high abundance of sequences from more than one eDNA marker and reported at all locations. We predict that the presence of these new species will be confirmed during future ecological surveys. Although we detected *S. vaginata*, its presence at RARE is dubious because it was found by a single eDNA marker (atpB‐rbcL‐184), at one location (3), and by a small number of reads (12). Further tests are required to confirm this finding.

The differences in the taxonomic assignment of OTUs using GenBank and the local reference library in BOLD (DS‐POTAM) are explained by two factors. First is the inconsistency between taxonomic treatments in European and North American literature (Kuzmina et al., 2017). For example, two species (*P. foliosus* and *P. pusillus*) are recognized in the Flora of North America (FNA; Haynes and Hellquist, 2000), whereas only one of these species (*P. pusillus*) is recognized in Europe (Uotila, 2006). We hypothesize that the specimen collected in Turkey potentially represents a *P. foliosus* population (the sequences MF694340 and KX273110 are identical) that was identified as *P. pusillus* (Aykurt et al., 2017) by European taxonomic treatments. The reference specimens for both species in the pondweed reference library were identified by the authors of the treatment in FNA (CCDB‐26261‐D06, CCDB‐26261‐B09) and are differentiated by our eDNA markers (Appendix S3). It allows us to be confident that the sequences detected in our eDNA samples correspond with the taxa identified as *P. foliosus* in the FNA. The second reason is an incompleteness of the reference library in GenBank for the taxa and/or the marker. Specifically, the lack of *atpB‐rbcL* sequences for the local species (*P. foliosus*) in GenBank resulted in the top hit match with a tropical species (*P. gayi*).

The developed method can be applied for the effective detection of aquatic plant species and to improve knowledge of their distribution. For example, *P. hillii* (Hill’s pondweed), a species of special concern in Ontario (COSEWIC, 2005), is known in the province only from several locations in Manitoulin Island, Bruce and Wellington counties, in the habitats associated with dolomitic limestone (Hellquist, 1984). It is possible that its range is wider than currently reported, as additional populations might occur in similar habitats along the Niagara Escarpment and the Precambrian contact line. Furthermore, a combination of the detected species of pondweeds can be used as a “fingerprint” for a biological community, or as an indicator of water quality, which is valuable to broader ecological and biomonitoring questions. Targeting a broader group of freshwater macrophytes through eDNA detection is an effective strategy for tracking underestimated plant diversity in aquatic habitats.

Our study demonstrated that eDNA can effectively detect different species of pondweeds in water samples, evaluate their potential under‐estimated diversity in aquatic communities, and identify locations of rare and protected species. The use of both chloroplast and nuclear markers improves species resolution among the selected species of pondweeds and increases reliability of the results for the species with comparably lower abundance. Finally, we demonstrated that the results of eDNA detection strongly depend on the completeness and accuracy of the reference libraries.

## DATA ACCESSIBILITY

The reference sequences were uploaded to BOLD (dx.doi.org/10.5883/DS‐POTAM) and GenBank (MF694321–MF694399, MF694493–MF694563).

## Supporting information

Appendix S1

Appendix S2

Appendix S3

Appendix S4

## References

[aps31155-bib-0001] APG, I. V. 2016 An update of the Angiosperm Phylogeny Group classification for the orders and families of flowering plants: APG IV. Botanical Journal of the Linnean Society 181: 1–20. https://doi.org/10.1111/boj.12385.

[aps31155-bib-0002] Aykurt, C. , J. Fehrer , D. Sari , Z. Kaplan , I. G. Deniz , E. Aydemir , and N. Imir . 2017 Hybridization between the linear‐leaved *Potamogeton* species in Turkey. Aquatic Botany 141: 22–28. https://doi.org/10.1016/j.aquabot.2017.05.005.

[aps31155-bib-0003] Barnes, M. A. , and C. R. Turner . 2016 The ecology of environmental DNA and implications for conservation genetics. Conservation Genetics 17: 1–17. https://doi.org/10.1007/s10592-015-0775-4.

[aps31155-bib-0004] Biggs, J. , N. Ewald , A. Valentini , C. Gaboriaud , T. Dejean , R. A. Griffiths , J. Foster , et al. 2015 Using eDNA to develop a national citizen science‐based monitoring programme for the great crested newt (*Triturus cristatus*). Biological Conservation 183: 18–28. https://doi.org/10.1016/j.biocon.2014.11.029.

[aps31155-bib-0005] Brouillet, L. , F. Coursol , S. J. Meades , M. Favreau , M. Anions , P. Bélisle , and P. Desmet . 2010 onward (continuously updated). VASCAN, the Database of Vascular Plants of Canada. Website http://data.canadensys.net/vascan/ [accessed 4 October 2016].

[aps31155-bib-0006] Caporaso, J. G. , J. Kuczynski , J. Stombaugh , K. Bittinger , F. D. Bushman , E. K. Costello , N. Fierer , et al. 2010 QIIME allows analysis of high‐throughput community sequencing data. Nature Methods 7: 335–336. https://doi.org/10.1038/nmeth.f.303. 20383131 10.1038/nmeth.f.303 PMC3156573

[aps31155-bib-0007] Chen, S. , H. Yao , J. Han , C. Liu , J. Song , L. Shi , Y. Zhu , et al. 2010 Validation of the ITS2 region as a novel DNA barcode for identifying medicinal plant species. PLoS ONE 5: e8613 https://doi.org/10.1371/journal.pone.0008613. 20062805 10.1371/journal.pone.0008613 PMC2799520

[aps31155-bib-0008] Cheng, T. , C. Xu , L. Lei , C. Li , Y. Zhang , and S. Zhou . 2016 Barcoding the kingdom Plantae: New PCR primers for ITS regions of plants with improved universality and specificity. Molecular Ecology Resources 16: 138–149. https://doi.org/10.1111/1755-0998.12438. 26084789 10.1111/1755-0998.12438

[aps31155-bib-0009] China Plant BOL Group . 2011 Comparative analysis of a large dataset indicates that internal transcribed spacer (ITS) should be incorporated into the core barcode for seed plants. Proceedings of the National Academy of Sciences USA 108: 19641–19646. https://doi.org/10.1073/pnas.1104551108. 10.1073/pnas.1104551108 PMC3241788 22100737

[aps31155-bib-0010] COSEWIC (Committee on the Status of Endangered Wildlife in Canada) . 2005 Assessment and status report on the Hill’s pondweed, Potamogeton hillii. COSEWIC Secretariat c/o Canadian Wildlife Service Environment Canada, Ottawa, Ontario, Canada.

[aps31155-bib-0011] COSEWIC (Committee on the Status of Endangered Wildlife in Canada) . 2007 Assessment and status report on the Ogden’s pondweed, Potamogeton ogdenii. COSEWIC Secretariat c/o Canadian Wildlife Service Environment Canada, Ottawa, Ontario, Canada.

[aps31155-bib-0012] Deiner, K. , H. M. Bik , E. Mächler , M. Seymour , A. Lacoursière‐Roussel , F. Altermatt , S. Creer , et al. 2017 Environmental DNA metabarcoding: Transforming how we survey animal and plant communities. Molecular Ecology 26: 5872–5895. https://doi.org/10.1111/mec.14350. 28921802 10.1111/mec.14350

[aps31155-bib-0013] Dejean, T. , A. Valentini , C. Miquel , P. Taberlet , E. Bellemain , and C. Miaud . 2012 Improved detection of an alien invasive species through environmental DNA barcoding: The example of the American bullfrog *Lithobates catesbeianus* . Journal of Applied Ecology 49: 953–959. https://doi.org/10.1111/j.1365-2664.2012.02171.x.

[aps31155-bib-0014] Desmet, P. , and L. Brouillet . 2013 Database of Vascular Plants of Canada (VASCAN): A community contributed taxonomic checklist of all vascular plants of Canada, Saint Pierre and Miquelon, and Greenland. PhytoKeys 25: 55–67. https://doi.org/10.3897/phytokeys.25.3100. 10.3897/phytokeys.25.3100 PMC3819130 24198712

[aps31155-bib-0015] Dibble, E. D. , and S. L. Harrel . 1997 Largemouth bass diets in two aquatic plant communities. Journal of Aquatic Plant Management 35: 74–78.

[aps31155-bib-0016] Dixon, M. H. , S. A. Hill , M. B. Jackson , R. G. Ratcliffe , and L. J. Sweetlove . 2006 Physiological and metabolic adaptations of *Potamogeton pectinatus* L. tubers support rapid elongation of stem tissue in the absence of oxygen. Plant Cell Physiology 47: 128–140. https://doi.org/10.1093/pcp/pci229. 16284407 10.1093/pcp/pci229

[aps31155-bib-0017] Edgar, R. C. 2010 Search and clustering orders of magnitude faster than BLAST. Bioinformatics 26: 2460–2461. https://doi.org/10.1093/bioinformatics/btq461. 20709691 10.1093/bioinformatics/btq461

[aps31155-bib-0018] Engel, S. 1988 The role and interactions of submersed macrophytes in a shallow Wisconsin lake. Journal of Freshwater Ecology 4: 329–341.

[aps31155-bib-0019] Environment Canada . 2015 Recovery strategy for Ogden’s Pondweed (Potamogeton ogdenii) in Canada. Environment Canada, Species at Risk Act Recovery Strategy Series, Ottawa, Ontario, Canada.

[aps31155-bib-0020] Fahner, N. A. , S. Shokralla , D. J. Baird , and M. Hajibabaei . 2016 Large‐scale monitoring of plants through environmental DNA metabarcoding of soil: Recovery, resolution, and annotation of four DNA markers. PLoS ONE 11: e0157505 https://doi.org/10.1371/journal.pone.0157505. 27310720 10.1371/journal.pone.0157505 PMC4911152

[aps31155-bib-0021] Fernald, M. L. 1932 The linear‐leaved North American species of *Potamogeton*, Section Axillaries *In* Memoirs of the Gray Herbarium of Harvard University, vol. 17 American Academy of Arts and Sciences, Cambridge, Massachusetts, USA.

[aps31155-bib-0022] Gleason, H. A. , and A. Cronquist . 1991 Manual of vascular plants of Northern United States and adjacent Canada. The New York Botanical Garden Press, Bronx, New York, USA.

[aps31155-bib-0023] Haynes, R. R. , and C. B. Hellquist . 2000 Potamogetonaceae *In* Flora of North America Editorial Committee [eds.], Flora of North America North of Mexico, vol. 22, 47–74. Oxford University Press, New York, New York, USA.

[aps31155-bib-0024] Hellquist, C. B. 1984 Observations of *Potamogeton hillii* in North America. Rhodora 86: 101–111.

[aps31155-bib-0025] Holmes, N. T. H. , P. J. Boon , and T. A. Rowell . 1998 A revised classification system for British rivers based on their aquatic plant communities. Aquatic Conservation: Marine and Freshwater Ecosystems 8: 555–578.

[aps31155-bib-0026] Ito, Y. , N. Tanaka , R. Pooma , and N. Tanaka . 2014 DNA barcoding reveals a new record of *Potamogeton distinctus* (Potamogetonaceae) and its natural hybrids, *Potamogeton distinctus* × *Potamogeton nodosus* and *Potamogeton distinctus* × *Potamogeton wrightii* (*Potamogeton × malainoides*) from Myanmar. Biodiversity Data Journal 2: e1073 https://doi.org/10.3897/BDJ.2.e1073. 10.3897/BDJ.2.e1073 PMC4030248 24855447

[aps31155-bib-0027] Ivanova, N. V. , A. J. Fazekas , and P. D. N. Hebert . 2008 Semi‐automated, membrane‐based protocol for DNA isolation from plants. Plant Molecular Biology Reporter 26: 186–198. https://doi.org/10.1007/s11105-008-0029-4.

[aps31155-bib-0028] Ivanova, N. , M. Kuzmina , and A. Fazekas . 2011 CCDB Protocols. Manual protocol employing centrifugation: glass fiber plate DNA extraction protocol for plants, fungi, echinoderms and mollusks. Available at http://ccdb.ca/site/wp-content/uploads/2016/09/CCDB_DNA_Extraction-Plants.pdf [accessed 7 December 2017].

[aps31155-bib-0029] Joshi, N. A. , and F. N. Fass . 2011 Sickle: A sliding‐window, adaptive, quality‐based trimming tool for FastQ files version 1.29. Available at https://github.com/najoshi/sickle [accessed 11 December 2017].

[aps31155-bib-0030] Katoh, K. , K. Misawa , K. Kuma , and T. Miyata . 2002 MAFFT: A novel method for rapid multiple sequence alignment based on fast Fourier transform. Nucleic Acids Research 30: 3059–3066. 12136088 10.1093/nar/gkf436 PMC135756

[aps31155-bib-0031] Kibbe, W. A. 2007 OligoCalc: An online oligonucleotide properties calculator. Nucleic Acids Research 35(Suppl 2): W43–W46. 17452344 10.1093/nar/gkm234 PMC1933198

[aps31155-bib-0032] Kuzmina, M. L. , T. W. A. Braukmann , A. J. Fazekas , S. W. Graham , S. L. Dewaard , A. Rodrigues , B. A. Bennett , et al. 2017 Using herbarium‐derived DNAs to assemble a large‐scale DNA barcode library for the vascular plants of Canada. Applications in Plant Sciences 5: 1700079 https://doi.org/10.3732/apps.1700079. 10.3732/apps.1700079 PMC5749818 29299394

[aps31155-bib-0033] Lindqvist, C. , J. De Laet , R. R. Haynes , L. A. A. Gesen , B. R. Keener , and V. A. Albert . 2006 Molecular phylogenetics of an aquatic plant lineage, Potamogetonaceae. Cladistics 22: 568–588. https://doi.org/10.1111/j.1096-0031.2006.00124.x. 10.1111/j.1096-0031.2006.00124.x 34892900

[aps31155-bib-0034] Lukacs, B. A. , G. Devai , and B. Tothmeresz . 2009 Aquatic macrophytes as bioindicators of water chemistry in nutrient rich backwaters along the Upper‐Tisza River (in Hungary). Phytocoenologia 39: 287–293. https://doi.org/10.1127/0340-269X/2009/0039-0287.

[aps31155-bib-0035] Lusa, M. G. , M. R. Torres Boeger , M. C. De Chiara Moço , and C. Bona . 2011 Morpho‐anatomical adaptations of *Potamogeton polygonus* (Potamogetonaceae) to lotic and lentic environments. Rodriguésia 62: 927–936.

[aps31155-bib-0036] Manen, J.‐F. , A. Natali , and F. Ehrendorfer . 1994 Phylogeny of Rubiaceae‐Rubieae inferred from the sequence of a cpDNA intergene region. Plant Systematics and Evolution 190: 195–211.

[aps31155-bib-0037] Marcel, M. 2011 Cutadapt removes adapter sequences from high‐throughput sequencing reads. EMBnet.journal 17: 10–12.

[aps31155-bib-0038] McNeill J., Turland N. J., Barrie F. R., Buck W. R., Greuter W., and Wiersema J. H. [eds.]. 2012 International code of nomenclature for algae, fungi, and plants. Koeltz Scientific Books, Konigstein, Germany.

[aps31155-bib-0039] Nielsen, K. M. , P. J. Johnsen , D. Bensasson , and D. Daffonchio . 2007 Release and persistence of extracellular DNA in the environment. Environmental Biosafety Research 6: 37–53. https://doi.org/10.1051/ebr:2007031. 17961479 10.1051/ebr:2007031

[aps31155-bib-0040] Parks Canada . 2012 Management plan for Hill’s pondweed (Potamogeton hillii) in Canada. Parks Canada Agency, Species at Risk Act Management Plan Series, Ottawa, Ontario, Canada.

[aps31155-bib-0041] Peng, K. , C. Luo , L. Luo , X. Li , and Z. Shen . 2008 Bioaccumulation of heavy metals by the aquatic plants *Potamogeton pectinatus* L. and *Potamogeton malaianus* Miq. and their potential use for contamination indicators and in wastewater treatment. Science of the Total Environment 392: 22–29. https://doi.org/10.1016/j.scitotenv.2007.11.032. 18178241 10.1016/j.scitotenv.2007.11.032

[aps31155-bib-0042] Price, M. N. , P. S. Dehal , and A. P. Arkin . 2010 FastTree 2—Approximately maximum‐likelihood trees for large alignments. PLoS ONE 5: e9490 https://doi.org/10.1371/journal.pone.0009490. 20224823 10.1371/journal.pone.0009490 PMC2835736

[aps31155-bib-0043] Ratnasingham, S. , and P. D. N. Hebert . 2007 BOLD: The Barcode of Life Data System (http://www.barcodinglife.org). Molecular Ecology Notes 7: 355–364. https://doi.org/10.1111/j.1471-8286.2007.01678.x. 18784790 10.1111/j.1471-8286.2007.01678.x PMC1890991

[aps31155-bib-0044] Robionek, A. , K. Banaś , R. Chmara , and J. Szmeja . 2015 The avoidance strategy of environmental constraints by an aquatic plant *Potamogeton alpinus* in running waters. Ecology and Evolution 5: 3327–3337. https://doi.org/10.1002/ece3.1598. 26380667 10.1002/ece3.1598 PMC4569029

[aps31155-bib-0045] Sandilands, A. 2005 Birds of Ontario: Habitat requirements, limiting factors and status. UBC Press, Vancouver, British Columbia, Canada.

[aps31155-bib-0046] Scriver, M. , A. Marinich , C. Wilson , and J. Freeland . 2015 Development of species‐specific environmental DNA (eDNA) markers for invasive aquatic plants. Aquatic Botany 122: 27–31.

[aps31155-bib-0047] Seymour, M. , I. Durance , B. J. Cosby , E. Ransom‐Jones , K. Deiner , S. J. Ormerod , J. K. Colbourne , et al. 2018 Acidity promotes degradation of multi‐species environmental DNA in lotic mesocosms. Communications Biology 1: 4 https://doi.org/10.1038/s42003-017-0005-3. 10.1038/s42003-017-0005-3 PMC6123786 30271891

[aps31155-bib-0048] Shaw, J. L. A. , L. Weyrich , and A. Cooper . 2016 Using environmental (e)DNA sequencing for aquatic biodiversity surveys: A beginner’s guide. Marine and Freshwater Research 68: 20–33. https://doi.org/10.1071/MF15361.

[aps31155-bib-0049] Sigsgaard, E. E. , H. Carl , P. R. Møller , and P. F. Thomsen . 2015 Monitoring the near‐extinct European weather loach in Denmark based on environmental DNA from water samples. Biological Conservation 183: 46–52. https://doi.org/10.1016/j.biocon.2014.11.023.

[aps31155-bib-0050] Tamura, K. , and M. Nei . 1993 Estimation of the number of nucleotide substitutions in the control region of mitochondrial DNA in humans and chimpanzees. Molecular Biology and Evolution 10: 512–526. https://doi.org/10.1093/oxfordjournals.molbev.a040023. 8336541 10.1093/oxfordjournals.molbev.a040023

[aps31155-bib-0051] Telfer, A. C. , M. R. Young , J. Quinn , K. Perez , C. N. Sobel , J. E. Sones , V. Levesque‐Beaudin , et al. 2015 Biodiversity inventories in high gear: DNA barcoding facilitates a rapid biotic survey of a temperate nature reserve. Biodiversity Data Journal 3: e6313 https://doi.org/10.3897/BDJ.3.e6313. 10.3897/BDJ.3.e6313 PMC4568406 26379469

[aps31155-bib-0052] Thiers, B. 2017 onward (continuously updated). Index Herbariorum: A global directory of public herbaria and associated staff. New York Botanical Garden’s Virtual Herbarium. Website http://sweetgum.nybg.org/science/ih/ [accessed 4 October 2016].

[aps31155-bib-0053] Ulloa, C. U. , P. Acevedo‐Rodríguez , S. Beck , M. J. Belgrano , R. Bernal , P. E. Berry , L. Brako , et al. 2017 An integrated assessment of the vascular plants species of the Americas. Science 358: 1614–1617. https://doi.org/10.1126/science.aao0398. 29269477 10.1126/science.aao0398

[aps31155-bib-0054] Uotila, P. 2006 to present. Potamogetonaceae. Euro+Med PlantBase: The information resource for Euro‐Mediterranean plant diversity. Website http://ww2.bgbm.org/EuroPlusMed/ [accessed 1 December 2017].

[aps31155-bib-0055] U.S. Fish and Wildlife Service . 2018 ECOS: Environmental Conservation Online System. Website https://ecos.fws.gov/ecp/ [accessed 14 May 2018].

[aps31155-bib-0056] Wetzel, R. G. 1983 Limnology, 2nd ed Saunders College Publishing, Philadelphia, Pennsylvania, USA.

[aps31155-bib-0057] White, T. J. , T. Bruns , S. Lee , and J. Taylor . 1990 Amplification and direct sequencing of fungal ribosomal RNA genes for phylogenetics *In* Innis M. A., Gelfand D. H., Sninsky J. J., and White T. J. [eds.], PCR protocols: A guide to methods and applications, 315–322. Academic Press, New York, New York, USA.

