# Supplementary material for: Finding the pond through the weeds: eDNA reveals underestimated diversity of pondweeds
Source: Appl Plant Sci. 2018 Jun 5;6(5):e01155. doi: 10.1002/aps3.1155 (PMC5991581; doi:10.1002/aps3.1155)
Supplement: Supplementary file 1 — Appendix S1 [file APS3-6-e01155-s001.docx]

**APPENDIX S1.** Primers and PCR conditions for Sanger and Ion Personal Genome Machine (Ion PGM) sequencing platforms.

**A. Primers**

| **Number** | **Primer names** | **Primer sequences (5′–3′)** | **In combination with** | **Mean length of amplicon (bp)** | **Pondweed specific/non-specific** | **Reference** |
| --- | --- | --- | --- | --- | --- | --- |
| 1 | atpB-2F | TATGAGAATCAATCCTACTACTTCT | 2 |  |  | Manen et al., 1994 |
| 2 | rbcL-2R |  | 1 |  |  | Manen et al., 1994 |
| 3 | ITS2-S2F | ATGCGATACTTGGTGTGAAT | 4 and 5 | 370 | No | Chen et al., 2010 |
| 4 | ITS2-S3R | GACGCTTCTCCAGACTACAAT | 3 | 475 | No | Chen et al., 2010 |
| 5 | ITS4 | TCCTCCGCTTATTGATATGC | 3 | ‒ | No | White et al., 1990 |
| 6 | atpB-110F | GAATGGAATCAATAAGGAAGTTAAC | 7 | 450 | Yes | Designed here |
| 7 | atpB-594R | TACTCTTTGATATGTATGGCGCAAC | 6 | ‒ | Yes | Designed here |
| 8 | ITS2-93F | TGCGTGGGCGTCAAGTTACGTT | 9 | 260 | Yes | Designed here |
| 9 | ITS-479R | AGCCTGACGTGGGGTCGC | 8 | ‒ | Yes | Designed here |
| 10 | atpB-134F | TAGAATTCGAACCTAAACTCG | 12 | 117 | Yes | Designed here |
| 11 | atpB-215F | GGAAGTATGTATCTTTTATCTATCA | 12 | 184 | Yes | Designed here |
| 12 | atpBR | CTCTTGACACTGATATACCT | 10 and 11 | ‒ | Yes | Designed here |
| 13 | ITS2-168F | CGACGAATGGTGGGTTGAAGTT | 14 | 157 | Yes | Designed here |
| 14 | ITS2R | AATCCAGCCTGACGTGGGGTCG | 13 | ‒ | Yes | Designed here |

**B. PCR conditions**

| **DNA region** | **Primers** | **PCR programs for Sanger and Ion PGM sequencing** |
| --- | --- | --- |
| **Sanger sequencing** | | |
| ITS2 | 1, 2, 3, 6, 7 | 98°C for 45 s; 35 cycles of 98°C for 10 s, 56°C for 30 s, 72°C for 40 s; final extension 72°C for 10 min |
| *atpB*-*rbcL* | 4,5 | 98°C for 45 s; 35 cycles of 98°C for 10 s, 52°C for 30 s, 72°C for 40 s; final extension 72°C for 10 min |
| **Ion PGM first round** | | |
| ITS2 | 8, 9 | 98°C for 45 s; 40 cycles of 98°C for 10 s, 56°C for 30 s, 72°C for 40 s; final extension 72°C for 10 min |
| *atpB*-*rbcL* | 10, 11, 12 | 98°C for 45 s; 40 cycles of 98°C for 10 s, 52°C for 30 s, 72°C for 40 s; final extension 72°C for 10 min |
| **Ion PGM second round** | | |
| ITS2 and *atpB-rbcL* | Fusion primers for UPGM | 98°C for 45 s; 20 cycles of 98°C for 10 s, 50°C for 30 s, 72°C for 40 s; final extension 72°C for 10 min |

**C. PCR cocktail per reaction**

| **PCR cocktail ingredients** | **Volume** |
| --- | --- |
| 5× buffer HF (with MgCl_2_) | 2 μL |
| 100% DMSO | 0.3 μL |
| ddH_2_0 | 6.32 μL |
| 10 μM forward primer | 0.1 μL |
| 10 μM reverse primer | 0.1 μL |
| 10 mM dNTPs | 0.056 μL |
| Phusion High Fidelity (5 U/μL) | 0.125 μL |
| DNA template | 1 μL per well |
| Total | 10 μL |

**D. Fusion primers for Ion PGM^a^**

| **Sample/eDNA marker with mean length of amplicon (bp)** | **atpB-rbcL-117** | **atpB-rbcL-184** | **ITS2-157** |
| --- | --- | --- | --- |
| Site 1 | atpBR-ion4/atpB-134F-trP1 | atpBR-ion1/atpB-215F-trP1 | ITS2R-ion7/ITS-168F-trP1 |
| Site 2 | atpBR-ion5/atpB-134F-trP1 | atpBR-ion2/atpB-215F-trP1 | ITS2R-ion8/ITS-168F-trP1 |
| Site 3 | atpBR-ion6/atpB-134F-trP1 | atpBR-ion3/atpB-215F-trP1 | ITS2R-ion9/ITS-168F-trP1 |
| DNA negative | atpBR-ion12/atpB-134F-trP1 | atpBR-ion10/atpB-215F-trP1 | ITS2R-ion14/ITS-168F-trP1 |
| PCR negative | atpBR-ion13/atpB-134F-trP1 | atpBR-ion11/atpB-215F-trP1 | ITS2R-ion15/ITS-168F-trP1 |

**^a^Fusion primer sequence (5′–3′):** *sequencing (or* ***P1*** ***ISP binding****) adapters /* key **/ molecular identifier (MID)** / primer

**atpBR-ion1:** *CCATCTCATCCCTGCGTGTCTCCGAC*TCAG**CTAAGGTAAC**CTCTTGACACTGATATACCT

**atpBR-ion2:** *CCATCTCATCCCTGCGTGTCTCCGAC*TCAG**TAAGGAGAAC**CTCTTGACACTGATATACCT

**atpBR-ion3:** *CCATCTCATCCCTGCGTGTCTCCGAC*TCAG**AAGAGGATTC**CTCTTGACACTGATATACCT

**atpBR-ion4:** *CCATCTCATCCCTGCGTGTCTCCGAC*TCAG**TACCAAGATC**CTCTTGACACTGATATACCT

**atpBR-ion5:** *CCATCTCATCCCTGCGTGTCTCCGAC*TCAG**CAGAAGGAAC**CTCTTGACACTGATATACCT

**atpBR-ion6:** *CCATCTCATCCCTGCGTGTCTCCGAC*TCAG**CTGCAAGTTC**CTCTTGACACTGATATACCT

**ITS2R-ion7:** *CCATCTCATCCCTGCGTGTCTCCGAC*TCAG**TTCGTGATTC** AATCCAGCCTGACGTGGGGTCG

**ITS2R-ion8:** *CCATCTCATCCCTGCGTGTCTCCGAC*TCAG**TTCCGATAAC** AATCCAGCCTGACGTGGGGTCG

**ITS2R-ion9:** *CCATCTCATCCCTGCGTGTCTCCGAC*TCAG**TGAGCGGAAC** AATCCAGCCTGACGTGGGGTCG

**atpBR-ion10:** *CCATCTCATCCCTGCGTGTCTCCGAC*TCAG**CTGACCGAAC**CTCTTGACACTGATATACCT

**atpBR-ion11:** *CCATCTCATCCCTGCGTGTCTCCGAC*TCAG**TCCTCGAATC**CTCTTGACACTGATATACCT

**atpBR-ion12:** *CCATCTCATCCCTGCGTGTCTCCGAC*TCAG**TAGGTGGTTC**CTCTTGACACTGATATACCT

**atpBR-ion13:** *CCATCTCATCCCTGCGTGTCTCCGAC*TCAG**TCTAACGGAC**CTCTTGACACTGATATACCT

**ITS2R-ion14:** *CCATCTCATCCCTGCGTGTCTCCGAC*TCAG**TTGGAGTGTC** AATCCAGCCTGACGTGGGGTCG

**ITS2R-ion15:** *CCATCTCATCCCTGCGTGTCTCCGAC*TCAG**TCTAGAGGTC** AATCCAGCCTGACGTGGGGTCG

**atpB-134F-trP1: *CCTCTCTATGGGCAGTCGGTGAT*** TAGAATTCGAACCTAAACTCG

**atpB-215F-trP1: *CCTCTCTATGGGCAGTCGGTGAT*** GGAAGTATGTATCTTTTATCTATCA

**ITS-168F-trP1: *CCTCTCTATGGGCAGTCGGTGAT*** CGACGAATGGTGGGTTGAAGTT
