# Supplementary material for: Finding the pond through the weeds: eDNA reveals underestimated diversity of pondweeds
Source: Appl Plant Sci. 2018 Jun 5;6(5):e01155. doi: 10.1002/aps3.1155 (PMC5991581; doi:10.1002/aps3.1155)
Supplement: Supplementary file 2 — Appendix S2 [file APS3-6-e01155-s002.docx]

**APPENDIX S2.** The *atpB-rbcL* and ITS2 GenBank accession numbers for the species of pondweeds (Potamogetonaceae) included in the pondweed DNA reference library (dx.doi.org/10.5883/DS-POTAM).

| **Species name** | ***atpB-rbcL* accession** | **ITS2 accession** |
| --- | --- | --- |
| *Potamogeton alpinus* | MF694534, MF694558, MF694524, MF694499 | MF694365, MF694380, MF694393, MF694356, JN999404 |
| *Potamogeton amplifolius* | MF694494, MF694560, MF694541 | MF694322, MF694375, MF694373 |
| *Potamogeton bicupulatus* | MF694559, MF694520 | MF694394, MF694351 |
| *Potamogeton confervoides* | MF694514, MF694506 | MF694325, MF694397, MF694398, MF694346, MF694337 |
| *Potamogeton crispus* | MF694547, MF694551, MF694495, MF694550 | MF694382, MF694329, MF694385, MF694324, KT695266 |
| *Potamogeton epihydrus* | MF694523, MF694517 | MF694355, MF694332, MF694348 |
| *Potamogeton foliosus* | MF694509 | MF694340, MF694352, MF694367 |
| *Potamogeton friesii* | MF694555 | MF694391, MF694369 |
| *Potamogeton gramineus* | MF694528, MF694552, MF694522, MF694563 | MF694387, MF694354, MF694399 |
| *Potamogeton hillii* | MF694549, MF694548, MF694525, MF694553 | MF694384, MF694383, MF694357, MF694388 |
| *Potamogeton illinoensis* | MF694513, MF694532 | MF694344, MF694363 |
| *Potamogeton natans* | MF694538 | MF694371 |
| *Potamogeton nodosus* |  | MF694335 |
| *Potamogeton oakesianus* | MF694512 | MF694343 |
| *Potamogeton obtusifolius* |  | MF694323, MF694395 |
| *Potamogeton perfoliatus* | MF694543, MF694527 | MF694377, MF694359 |
| *Potamogeton praelongus* | MF694511, MF694561, MF694540 | MF694342, MF694396, MF694374 |
| *Potamogeton pulcher* | MF694519 | MF694350 |
| *Potamogeton pusillus* | MF694537, MF694504, MF694510, MF694542, MF694539 | MF694347, MF694370, MF694334, MF694341,MF694345, MF694376, MF694372 |
| *Potamogeton richardsonii* | MF694507, MF694556, MF694536, MF694515 | MF694338, JN999405, MF694368, JN999406 |
| *Potamogeton robbinsii* | MF694521, MF694493 | MF694353, MF694321 |
| *Potamogeton spirillus* | MF694531, MF694554 | MF694360, MF694362, MF694339, MF694389, MF694390 |
| *Potamogeton strictifolius* | MF694557 | MF694392, MF694386 |
| *Potamogeton subsibiricus* | MF694497, MF694535 | MF694327, MF694366 |
| *Potamogeton vaseyi* | MF694505, MF694530 | MF694336, MF694361 |
| *Potamogeton zosteriformis* | MF694501, MF694518, MF694533, MF694496 | MF694331, MF694349, MF694364, MF694326 |
| *Stuckenia filiformis* | MF694508, MF694529, MF694545, MF694502, MF694544, MF694516 | JN999660, JN999658, MF694379, JN999659, MF694378 |
| *Stuckenia pectinata* | MF694562, MF694498 | KT695300, MF694328 |
| *Stuckenia vaginata* | MF694546, MF694503, MF694500 | MF694381, MF694333, MF694330 |
| *Zannichellia palustris* | MF694526 | MF694358 |
