# Supplementary material for: Finding the pond through the weeds: eDNA reveals underestimated diversity of pondweeds
Source: Appl Plant Sci. 2018 Jun 5;6(5):e01155. doi: 10.1002/aps3.1155 (PMC5991581; doi:10.1002/aps3.1155)
Supplement: Supplementary file 4 — Appendix S4 [file APS3-6-e01155-s004.docx]

**APPENDIX S4.** The number of dereplicated sequences (FASTX Toolkit: http://hannonlab.cshl.edu/fastx_toolkit/) and operational taxonomic units (OTUs) (Uclust v1.2.22q: Edgar, 2010) associated with three eDNA markers (atpB-rbcL-117, atpB-rbcL-184, and ITS2-157) for three locations and a negative PCR control.

| **Marker** | **Location and negative control** | **No. of dereplicated sequences** | **No. of OTUs** |
| --- | --- | --- | --- |
| **atpB-rbcL-117** | 1 | 21,233 | 272 |
| **atpB-rbcL-184** | 1 | 3731 | 58 |
| **ITS2-157** | 1 | 91,236 | 11,422 |
| **atpB-rbcL-117** | 2 | 8210 | 150 |
| **atpB-rbcL-184** | 2 | 5535 | 56 |
| **ITS2-157** | 2 | 74,864 | 9491 |
| **atpB-rbcL-117** | 3 | 3412 | 70 |
| **atpB-rbcL-184** | 3 | 4336 | 57 |
| **ITS2-157** | 3 | 42,285 | 4333 |
| **atpB-rbcL-184** | Negative PCR | 4 | 1 |
